# Vergence and Strabismus in Neurodegenerative Disorders

**DOI:** 10.3389/fneur.2018.00299

**Published:** 2018-05-16

**Authors:** Sarah L. Kang, Aasef G. Shaikh, Fatema F. Ghasia

**Affiliations:** ^1^Case Western Reserve University School of Medicine, Cleveland, OH, United States; ^2^Daroff-Dell’Osso Ocular Motility Laboratory, Louis Stokes Cleveland VA Medical Center, Cleveland, OH, United States; ^3^Cole Eye Institute, Cleveland Clinic, Cleveland, OH, United States

**Keywords:** strabismus, diplopia, neurodegenerative, Parkinson’s disease, spinocerebellar ataxia, Machado–Joseph disease

## Abstract

Maintaining proper eye alignment is necessary to generate a cohesive visual image. This involves the coordination of complex neural networks, which can become impaired by various neurodegenerative diseases. When the vergence system is affected, this can result in strabismus and disorienting diplopia. While previous studies have detailed the effect of these disorders on other eye movements, such as saccades, relatively little is known about strabismus. Here, we focus on the prevalence, clinical characteristics, and treatment of strabismus and disorders of vergence in Parkinson’s disease, spinocerebellar ataxia, Huntington disease, and multiple system atrophy. We find that vergence abnormalities may be more common in these disorders than previously thought. In Parkinson’s disease, the evidence suggests that strabismus is related to convergence insufficiency; however, it is responsive to dopamine replacement therapy and can, therefore, fluctuate with medication “on” and “off” periods throughout the day. Diplopia is also established as a side effect of deep brain stimulation and is thought to be related to stimulation of the subthalamic nucleus and extraocular motor nucleus among other structures. In regards to the spinocerebellar ataxias, oculomotor symptoms are common in many subtypes, but diplopia is most common in SCA3 also known as Machado–Joseph disease. Ophthalmoplegia and vergence insufficiency have both been implicated in strabismus in these patients, but cannot fully explain the properties of the strabismus, suggesting the involvement of other structures as well. Strabismus has not been reported as a common finding in Huntington disease or atypical parkinsonian syndromes and more studies are needed to determine how these disorders affect binocular alignment.

## Introduction

Proper alignment and coordination of the eyes is essential for accurately perceiving the visual environment. Because the eyes are separated in space and thus receive different images, fine ocular motor control is required in order to reconcile this disparity and achieve a cohesive image. This is done in part *via* sensory fusion, which is a cortical neurological process by which the cortex perceives the two retinal images as one. There is a level of normal disparity that is tolerated by the cortex, referred to as the fixation disparity (1). Within this visual angle, also known as Panum’s area, the cortex is able to achieve visual fusion and process the two distinct images as one. Panum’s area is transiently exceeded when subjects make head movements (2) or under real-world natural viewing conditions (3, 4) with no perception of diplopia. However, when the cortex is unable to achieve sensory fusion, extraocular vergence movements work to bring the eyes within the bounds of Panum’s area to permit fusion. Thus, the vergence system is an important component of ocular motor control and is essential for achieving a coherent visual image. Vergence eye movements can be broadly divided into two categories: fusional, which is stimulated by a disparity between the retinal images as discussed above, and accommodative, which works alongside accommodation of the lens and pupil to correct the visual blur.

**Figure 1:**
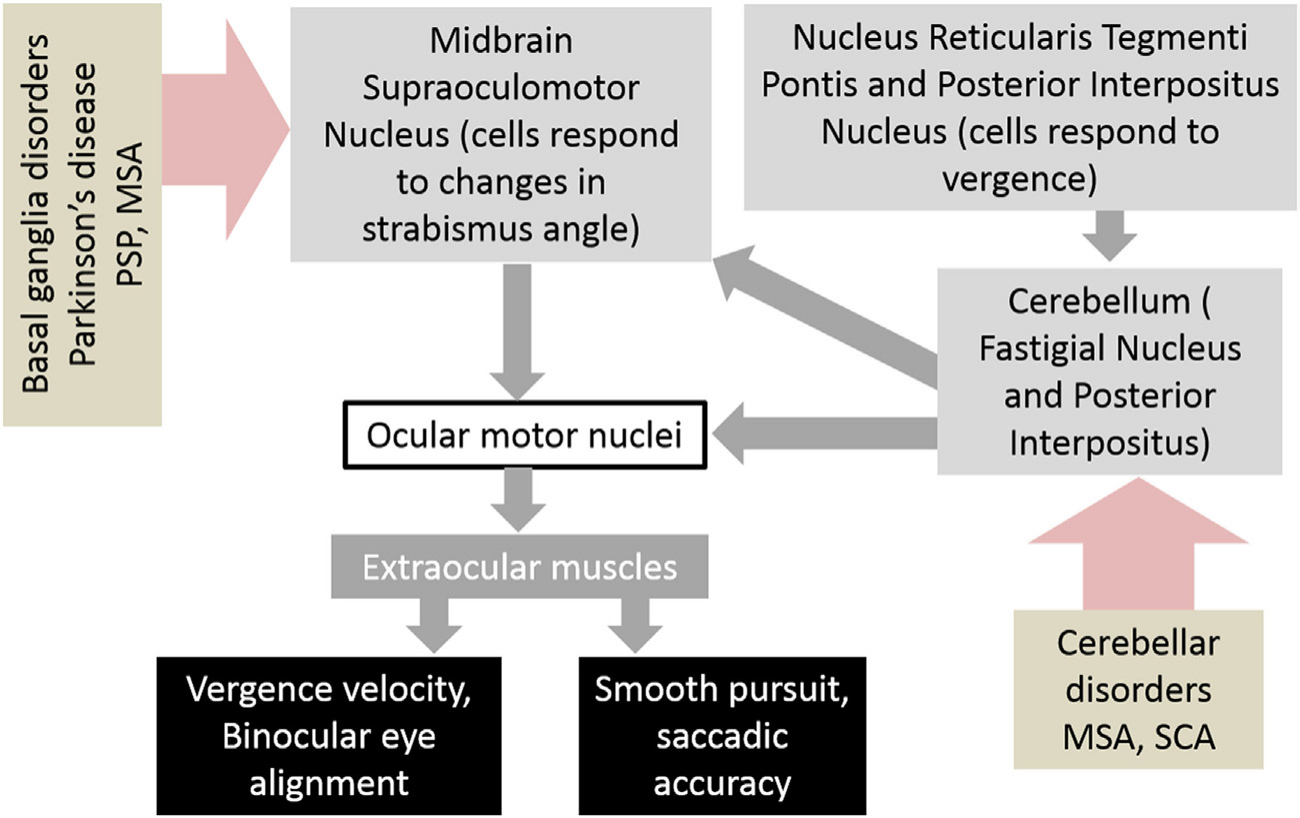
Schematic of neural circuits that result in abnormalities of vergence and strabismus in basal ganglia and cerebellar disorders. Abbreviations: PSP, progressive supranuclear palsy; MSA, multiple system atrophy; SCA, spinocerebellar ataxia.

The neuroanatomy of the vergence system has been the subject of much research and discussion. Understanding the neuroanatomical substrates involved in vergence aids in understanding how these pathways are affected by disease and how they interact with other ocular motor networks such as saccades. The generation of vergence commands starts with premotor commands, which are generated in the brainstem and then transmitted *via* ocular motor neurons to the extraocular muscles. The areas of the brainstem that have been found to be involved in vergence movements are the midbrain supraoculomotor area, the medial longitudinal fasciculus (MLF), and the paramedian pontine reticular formation (PPRF). The midbrain supraoculomotor area contains neurons that control slow extraocular muscle fibers involved in vergence (5–7), with different neurons responsible for vergence velocity and angle (8, 9). Increased activation of this region of the midbrain has been demonstrated by fMRI during vergence movements (10). On the other hand, the MLF is thought to carry signals that inhibit vergence, evidenced by studies of induced acute internuclear ophthalmoplegia in primates (11–13). The PPRF contains premotor burst neurons that play a role in controlling horizontal saccades and vergence movements; together these help generate gaze shifts in 3-D space (14, 15). Next, after the premotor commands have been generated in the brainstem, ocular motor neurons carry the commands to the extraocular muscles that carry out the movements. These ocular motor neurons are divided into four subgroups A–D within the oculomotor nucleus, with subgroup C believed to be most closely involved with the generation of slow eye movements such as vergence (6, 16). It is believed that outflow of the basal ganglia affects the brainstem network responsible for binocular control. As a result, an impairment in the basal ganglia outflow, as expected in degenerative forms of neurological disorders such as parkinsonism, can lead to abnormal binocular control (Figure 1).

The cerebellum is also involved in vergence, although its exact role is unclear. Evidence for this is seen in patients with acute cerebellar lesions who exhibit convergence insufficiency (17), and also the observation that ablation of the cerebellum in monkeys causes transient paralysis of vergence (18). Functional imaging also demonstrates activation of the cerebellar hemispheres and vermis during the near response (19) and while performing a binocularity discrimination task (20). Figure 1 depicts a schematic diagram of the neural substrate responsible for vergence eye movements and how disorders of basal ganglia and cerebellum affect them.

Under real-world viewing conditions, vergence movements almost always occur with saccadic eye movements to account for rapid shifts in space and depth (21, 22). For instance, an approaching target that is moving across the field of vision, rather than just directly head-on, will require horizontal saccades in addition to vergence to correctly track the object. Thus, it is important to consider how the neural networks for these two types of eye movements interact. A vergence integrator has been proposed to explain how the eyes maintain their vergence position at the end of saccadic eye movements (23). Much like the neural integrator for gaze holding, the vergence integrator is thought to receive signals from vergence burst cells and combine information about vergence velocity and position (24). While this vergence integrator is conceptually separate from the gaze holding integrator, there is evidence that suggests that they send signals over the same neural networks (25–27). Vergence movements are faster when they occur with saccades; an increase in saccadic peak velocity corresponds with an increase in the vergence peak velocity (28, 29). To explain this correspondence, it has been proposed that parallel saccadic and vergence pathways both receive input from omnipause neurons (30). However, this alone is not sufficient to explain the proportionate changes in velocity. One potential explanation is that the saccadic drive amplifies vergence motor error signals (29). However, an exception to this is seen when subjects are asked to make a shift from a far target to a near target that is higher. Under these viewing conditions, which are a relatively rare occurrence in nature, the vergence peak velocity is delayed from the saccadic peak velocity by about 100 ms (31). In conclusion, the vergence and saccadic systems are conceptually distinct, but interact with one another when they occur at the same time.

Phoria adaptation is another aspect of oculomotor control that interacts with vergence. Phoria is defined as the relative deviation of the visual axes of the two eyes that occurs when a single target is viewed with one eye. For example, if a wedge prism is placed in front of one eye, the subject’s phoria changes with the prismatic demand (32). Phoria also responds to the vestibulo-ocular reflex (33) and accommodation (34, 35), which provide different contexts for adaptation of vergence. Adjustments in phoria can be thought of as changes in tonic vergence. Tonic vergence is the product of fast and slow fusional systems (36, 37). Both are leaky integrators, in which the fast fusional system has a time constant of seconds compared to minutes in the slow fusional system (34, 38). Of note, individuals with convergence insufficiency have been demonstrated to have impaired prism adaptation in the horizontal (but not vertical) plane (39, 40). This supports the evidence that phoria adaptation and vergence movements are closely related. However, the neuroanatomical substrates of phoria adaptation are not completely understood. There is likely some overlap with the neuroanatomical structures involved in vergence described above. Studies in primates have demonstrated the importance of the midbrain vergence-related neurons in carrying phoria signals (41). The role of the cerebellum in phoria adaptation is somewhat controversial, and some studies have shown impairment in phoria adaptation in patients with cerebellar lesions (42, 43) while other studies show no effect of cerebellar lesion on phoria adaptation (44). Lesions made in the dorsal vermis in monkeys impaired binocular movements, including phoria adaptation (45).

Considering the anatomical dispersion of these neural networks, it is not surprising that they are often affected by neurodegenerative disease. These disorders, including Parkinson’s disease, atypical parkinsonism, spinocerebellar ataxias (SCA), and Huntington disease, have diverse effects on motor and cognitive function. Many ocular motor effects have been well-documented and can even aid in the diagnosis of disorders that have characteristic eye movement abnormalities (46, 47). Furthermore, these deficits have been shown to have a significant negative impact on vision-related quality of life (48, 49). Discussion of eye movement abnormalities in disease has been focused primarily on voluntary movements, such as saccades; however, there is a paucity of literature discussing the effects of neurodegeneration on binocular alignment. Thus, this review will address strabismus as a disorder of ocular alignment and vergence in neurodegenerative disorders affecting the motor system, such as the Parkinson’s disease and SCA.

Strabismus is defined as a misalignment of the eyes and can result in disorienting diplopia, loss of depth perception, and the negative social impact and a higher rate of symptoms related to depression and anxiety (50). Strabismus is present in an estimated 2–4% of children, an incidence that decreases significantly with age (51, 52). The etiology of strabismus may be broadly divided into congenital and acquired categories. Although it is commonly congenital, acquired strabismus may be a sign of a more serious underlying condition. In elderly individuals, strabismus is commonly found as an ocular manifestation of various neurodegenerative disorders. This review will focus on vergence abnormalities and strabismus as it appears in Parkinson’s disease, atypical parkinsonism, Huntington disease, and SCA. We will discuss clinical features as they relate to these disorders and their utility in diagnosis and tracking disease progression, as well as their response to treatment. Finally, we will discuss the underlying neural pathways behind these findings and their significance in disease pathology. The neurodegenerative disorders also present with other ocular motor deficits; although they are not discussed in detail, Table 1 provides a summary.

**Table 1:** Eye movement abnormalities in neurodegenerative disorders.

Disorder	Oculomotor findings
Parkinson’s disease	Increased saccade latency, decreased saccade amplitude, increased anti-saccade error rate (53, 55, 56) Diplopia in up to 20% (58) Convergence insufficiency (57) Increased convergence and divergence latency (60)
SCA3	Ophthalmoplegia, lid retraction, diplopia (74, 77) Strabismus in up to 83% (78) Divergence insufficiency (77)
SCA6	Diplopia in up to 50% (84) Vertical nystagmus, horizontal gaze-evoked nystagmus (85)
Huntington disease	Increased saccade latency, decreased saccade velocity, increased anti-saccade error rate (91–94)
Multiple system atrophy	Blepharospasm, square-wave jerks (97) Rare reports of vergence paresis resulting in diplopia (99)
Progressive supranuclear palsy	Slow vertical saccades, vertical gaze palsy (100) Square-wave jerks (101)
Corticobasal degeneration	Asymmetric saccadic apraxia, increased saccade latency, increased anti-saccade error (106, 107)
Dementia with Lewy bodies	Increased saccadic latency, increased anti-saccade error rate (110, 111) Case reports of supranuclear gaze palsy (112)

## Saccades, Vergence, and Strabismus in Parkinson’s Disease

Parkinson’s disease is a progressive neurological disorder characterized by loss of dopaminergic neurons in the substantia nigra, interfering with the dopamine signaling pathways of the basal ganglia and resulting in the classic constellation of tremor, bradykinesia, and postural instability. Growing evidence shows that the motor symptoms of Parkinson’s disease also extend to eye movements. Visual and ocular motor disturbances may be more common than previously thought and can have a significant impact on an individual’s quality of life and ability to navigate their surroundings. A study of 27 Parkinson’s disease patients revealed a significantly lower composite Visual Function Questionnaire (VFQ) score compared to healthy controls (87.1 ± 8.69 vs. 96.6 ± 3.05), including lower scores on almost every subscale, most notably those for near vision and ocular motor function (48). Specifically, patients with Parkinson’s disease display increased saccade latency and decreased amplitude of saccades, requiring a greater number of saccades to reach the desired target, and displaying more frequent errors during anti-saccade tasks (53–55). These findings seem to suggest that the classically observed motor findings of difficulty initiating movement and carrying out smooth repetitive movements, i.e., the small shuffling steps of a Parkinson’s patient, extend to saccadic eye movements as well.

Strabismus may present in Parkinson’s disease as a nonspecific complaint such as double vision (diplopia) or difficulty reading. One study of 39 Parkinson’s disease patients reported tired or blurred eyes while reading (*n* = 9, 23.1%) and diplopia (*n* = 3, 7.7%) as the most common visual complaints. Other studies report diplopia in 18–20% of Parkinson’s disease patients (56, 57), and all subjects in a study of 44 Parkinson’s disease patients with diplopia also had convergence insufficiency (56). The prevalence of strabismus in Parkinson’s disease suggests that dopamine may play a role in vergence pathways, and that disruption of the vergence system in Parkinson’s disease may be more common than previously thought.

A study of vergence eye movements in 18 Parkinson’s disease patients using video-oculography found significantly increased latency for both convergence and divergence movements in the horizontal and vertical planes, compared to healthy controls. Decreased velocity and gain were also described, but only for divergence movements in the vertical plane (58). These findings are consistent with previous studies in primates showing that separate areas in the brain control convergence and divergence, and that the midbrain supraoculomotor area plays a large role in controlling vergence movements (5, 59). The mesencephalic reticular formation, which is involved in mediating the velocity of vergence eye movements, is complemented by a separate group of convergence burst cells located in the dorsal mesencephalic region, rostral to the superior colliculus (24). It is possible that a more robust neural network is in place for mediating convergence eye movements, enabling them to compensate for motor insufficiency in Parkinson’s disease.

As discussed above, the vergence and saccadic oculomotor pathways interact whenever these movements occur at the same time. Thus, disorders of vergence in Parkinson’s disease may be the result of direct effects of the disease on vergence motor control, coupled with disturbances in the saccadic pathway indirectly leading to effects on vergence. Saccadic dysfunction has been well documented in Parkinson’s disease, thus it should be unsurprising that vergence abnormalities are common as well.

### Response to Treatment

Convergence insufficiency has been shown to improve upon administration of levodopa (60) and with deep brain stimulation (DBS) in conjunction with levodopa/carbidopa (48). In the previously mentioned study of 27 Parkinson’s patients, the convergence amplitude improved in the “on” phase of medication compared to the “off” phase (14.8 ± 10.3 vs. 10.7 ± 9.0), although it was still significantly worse than healthy controls (24.1 ± 8). Similarly, the near point of convergence improved in the “on” phase compared to the “off” phase (13.1 ± 9.1 vs. 18.1 ± 12.2), but was still more remote than controls (8.7 ± 4.5). However, although most subjects exhibited substantial exotropia at near, there was no difference in the mean exodeviation or ocular ductions with medication on/off periods (48). The fact that convergence ability fluctuates with dopamine dosage throughout the day presents a particular challenge in the ophthalmic management of these patients and may contribute to the negative impact on vision-related quality of life. Timing with medication should be considered when performing an ophthalmologic exam on PD patients.

### Strabismus Following DBS

Dystonia and eye deviation are well-documented side effects of subthalamic nucleus (STN) stimulation, the most commonly targeted structure in DBS surgery (61). Patients undergoing DBS surgery can develop transient diplopia that usually resolves after reprogramming the stimulation parameters; diplopia was observed in 2 of a study of 79 patients receiving DBS (2.5%) (62, 63). The diplopia is likely related to the direct, high frequency stimulation of the STN and surrounding structures, such as the corticospinal and corticobulbar tracts as they pass through the internal capsule, lateral to the STN. The suprabulbar fibers of the extraocular motor nerve or nuclei may also be affected, as fibers pass along the border of the red nucleus and may be affected by implants placed too far medially (61).

A case of hypertropia resulting in vertical diplopia was reported in a Parkinson’s disease patient following DBS implantation, although this was due to hemorrhage at the site of implantation and not the stimulation itself (64). Strabismus has also been reported as a side effect of DBS of the medial forebrain bundle as a treatment for depression; this strabismus was only present at high currents and could be rapidly resolved by adjusting the stimulation parameters (65, 66). Strabismus and diplopia are established side effects of DBS, and patients should be monitored for these conditions post-operatively to ensure that these symptoms do not interfere with quality of life and to rule out underlying structural abnormalities that can arise as surgical complications.

### Strabismus as a Biomarker

Examining the qualities of strabismus and vergence characteristics in Parkinson’s disease offers insight into disease pathophysiology and explores the question of whether these findings are useful as biomarkers of disease progression. A study of 39 patients with Parkinson’s disease examined the correlation between ocular abnormalities and duration and severity of disease (67). Visual complaints, most commonly convergence insufficiency, were more common in patients with Parkinson’s disease than healthy controls (12/39 vs. 0/39). When Parkinson’s disease patients were stratified based upon duration of disease, there was no significant difference in the rates of ocular findings; however, there was a significant correlation between severity of disease and frequency of visual complaints. Thus, vergence insufficiency may be useful as a measure of disease severity and quality of life independent of disease duration.

The response of convergence insufficiency to conventional Parkinson’s treatment supports its correlation with overall disease pathophysiology and symptomatology. While these ocular findings are neither necessary nor sufficient for a diagnosis of Parkinson’s disease and are less useful than the existing diagnostic criteria in this regard, their correlation with overall severity of disease and the fact that they may be quantitatively and noninvasively measured in the clinic offers a promising biomarker for tracking disease progression. More studies are required to establish their reliability and reproducibility as biomarkers.

## Saccades, Vergence, and Strabismus in Spinocerebellar Ataxia

The SCA are a heterogeneous group of disorders characterized by polyglutamine repeats, resulting in cerebellar ataxia and degeneration of structures, such as the basal ganglia, brainstem, dorsal columns and ventral horn of the spinal cord, and peripheral nerves (68–71). Although the precise role of the cerebellum in vergence is unclear, the cerebellar lesions in primates cause transient vergence paralysis (18). Additional symptoms, such as nystagmus, slow saccades, extrapyramidal signs, and tremor, are associated with various types of SCA, depending on the location of the genetic abnormality. At least 40 types have been identified to date, of which 28 have an identified pathogenic gene (72).

Ocular findings in SCA are common and have a negative impact on vision-related quality of life. A study of 19 SCA patients found significantly decreased scores on VFQ in regards to general vision, near vision, distance vision, driving, peripheral vision, and overall composite score compared to the general population (49). Like many other trinucleotide repeat disorders, symptom severity and age of onset vary with the size of the repeat expansion, and it is expected that ocular findings follow this pattern. Unlike Parkinson’s disease, in which ocular findings are usually not specific enough to be sufficient for diagnosis, certain types of SCA have characteristic ocular findings, which may aid in guiding the diagnosis of a particular type of SCA. An excellent summary of characteristic ocular findings in various SCAs may be found in Leigh and Zee’s Neurology of Eye Movements (73).

SCA3, also known as Machado–Joseph disease (MJD), is the most common of the autosomal dominant SCA (74). SCA3 is caused by a mutation in the SCA3/MJD gene on chromosome 14q32, which encodes the ataxin 3 protein (75). Characteristic findings include ophthalmoplegia, diplopia, lid retraction resulting in a “staring” or “bulging eye” appearance, facial fasciculations, spasticity, muscle fasciculations, and severe hyper- or hyporeflexia (74). Diplopia has been found to be more common in SCA3 than the other SCAs (74, 76). A study of 12 SCA3 patients found strabismus in 10 individuals (83%) (77). The prevalence of strabismus in SCA3 invites consideration of the underlying mechanism and pathways affected.

The study of one Japanese family with SCA3 found that this diplopia was the result of impaired divergence, which manifested itself as double vision that worsened when looking at distant targets but improved on lateral gaze (as opposed to an abducens palsy in which diplopia would be expected to worsen on lateral gaze) (76). Another study of seven patients with adult-onset esotropia found the esotropia to be of cerebellar origin, despite an initial misdiagnosis as lateral rectus paresis (78). These studies suggest that diplopia may be an early sign of cerebellar dysfunction. Cerebellar dysfunction has been implicated in increased convergence tone (79), offering a possible cerebellar pathophysiology for strabismus in patients with SCA. In addition, MRI and pathological studies of SCA3 patients have found significant atrophy of the brainstem and cerebellar vermis corresponding with the size of the trinucleotide repeat expansion in SCA3, particularly affecting the pontine reticular formation, but with relative sparing of the oculomotor, trochlear, and abducens nuclei (80, 81). These findings differentiate the pathophysiology of strabismus in SCA3 from an oculomotor or abducens nerve palsy, suggesting that the primary mechanism of strabismus is not ophthalmoplegia, but rather the lesion occurs higher in the vergence command pathway with the generation of premotor commands in the brainstem and cerebellum.

While it is possible that both vergence impairment and ophthalmoplegia may be present, the severity and incidence of the diplopia does not correspond to the severity of ophthalmoplegia (82), suggesting that ophthalmoplegia alone is not solely responsible for the ocular findings in SCA3. In the previously mentioned study of 12 SCA3 patients, those with exotropia had no distance-near disparity, and no patients had esotropia that worsened at distance, suggesting the absence of divergence insufficiency in this patient sample. Overall, the properties of strabismus in half of the strabismus patients in the study could not be explained by co-existing ophthalmoplegia and vergence abnormalities, suggesting involvement of structures above and beyond the vergence pathways, such as the midbrain, deep cerebellar nuclei, and superior cerebellar peduncle (77).

Diplopia has also been reported in up to 50% of patients with SCA6 (83). Downbeat nystagmus is considered a characteristic ocular finding for SCA6, as it was found in 84% of SCA6 patients compared to 5.2% of patients with other forms of SCA (84). Although there is less evidence describing the underlying pathophysiology of the strabismus in these patients, it is likely that a similar combination of ophthalmoplegia, vergence insufficiency, and other structures are involved.

### Response to Treatment

Given that treatment of SCAs is mostly supportive with little in the form of disease-modifying drugs, not much is known about the response of strabismus to treatment in these disorders. A recent randomized trial of varenicline, a nicotinic acetylcholine receptor partial agonist used in smoking cessation, in 20 patients with SCA3 demonstrated improvement in gait, rapid alternating movements, and timed 25-foot walk (85). However, eye movements and vision-related outcomes were not measured as part of the study. It is possible that improved motor control in gait and rapid alternating movements will also be reflected in ocular motor control, although this is yet to be confirmed. Another recent study evaluating the use of nerve growth factor as a treatment for 21 patients with SCA3 also demonstrated improvements in ataxia (65), particularly in subsections on stance, speech, finger chase, rapid alternating movements, and heel-to-shin (86). While these studies suggest promising potential treatments for SCA, more thorough study is needed. Given that oculomotor findings feature prominently in several SCA subtypes, including eye movement and vision-related outcomes in studies of potential SCA treatments would offer additional insight into the impact of treatment on disease pathophysiology and quality of life.

## Saccades, Vergence, and Strabismus in Huntington Disease

Huntington disease is an autosomal dominant neurodegenerative disorder caused by a trinucleotide repeat expansion in the huntingtin gene (87). Characteristic symptoms include choreiform movements, dystonia, hyperreflexia, and dementia (88, 89). Ophthalmologic symptoms have also been reported; specifically, saccade latency is increased along with anti-saccade error rate and impaired ability to suppress saccades (90–93). In contrast, vestibulo-ocular reflex and smooth pursuit movements are relatively preserved until late into the disease (93, 94). Of note, a slight increase in saccade latency and a decreased number of memory-guided saccades were found in presymptomatic Huntington gene carriers compared to non-gene carriers, suggesting that oculomotor control in Huntington could serve as an early biomarker (95).

While saccades are certainly affected in Huntington disease and may potentially serve as a biomarker for detection of symptoms and tracking disease progression, little is known about how Huntington disease affects vergence control. Diplopia is rarely reported in Huntington patients, suggesting that this is not usually a prominent finding. Further study may be warranted into how Huntington disease affects binocular fusion, if at all, or if there is some disconjugacy of saccades that may reflect a disruption of binocular ocular motor control.

## Saccades, Vergence, and Strabismus in Atypical Parkinsonian Syndromes

Multiple system atrophy (MSA), progressive supranuclear palsy (PSP), corticobasal degeneration (CBD), and dementia with Lewy bodies (DLB) are examples of atypical parkinsonian syndromes. That is, parkinsonian motor features are included in their constellation of symptoms, although the fundamental pathophysiology may differ. Since there is an overlap of symptoms, it is not unreasonable to expect that many of the ocular motor findings seen in Parkinson’s disease would be seen in atypical parkinsonism as well.

### Multiple System Atrophy

Multiple system atrophy is characterized by parkinsonism, ataxia, and autonomic dysfunction (96). It can be broken down into three types: parkinsonian, in which parkinsonian symptoms are predominant, cerebellar, in which cerebellar symptoms such as impaired coordination and speech are predominant, and combined, which has features of both types.

Given the similarities between Parkinson’s disease and MSA, one might expect diplopia to also feature prominently in MSA. One study of 20 patients with MSA found that reading speed was mildly affected, but no diplopia was reported (97). A case study described two MSA patients with diplopia that was the result of vergence paresis, with no signs of abducens palsy (98). However, a recent study of 39 MSA patients identified conjugate eye movement abnormalities in 33% of patients and ocular misalignment in another 18%. Additionally, the presence of ocular findings was correlated with a shorter time from diagnosis to death (99). These more recent findings suggest that abnormalities of eye alignment are more prevalent in MSA than previously known and also that they correlate with a poorer prognosis. More study would be worthwhile to further characterize these findings and explore their potential as biomarkers of disease progression and prognosis. Unlike in Parkinson’s disease, patients with MSA have a variable response to levodopa/carbidopa therapy (100). Little is known about how these treatments affect vision and oculomotor control.

### Progressive Supranuclear Palsy

As the name suggests, PSP is characterized by parkinsonism plus gaze palsies. Although the disease primarily and initially affects eye movement in the vertical direction, it can progress to involve horizontal saccades as well and develop into complete ophthalmoplegia (101). A common eye movement finding in PSP is square-wave jerks, which are saccadic intrusions that occur during attempted fixation (102).

A case report published in 2009 described a case of PSP that had horizontal diplopia as its presenting symptom, thought to be due to vergence abnormalities from degenerative effects on midbrain nuclei (103). It is interesting that this individual presented with horizontal gaze abnormalities, although he did go on to develop slowing of vertical saccades and square-wave jerks as is typical in PSP. The proximity of midbrain structures responsible for controlling vergence and horizontal and vertical saccades could explain this presentation, as this area of the midbrain is heavily affected by tau pathology in PSP (104). More studies are needed to determine exactly how common vergence abnormalities and diplopia are in PSP, although the proposed pathology suggests that these structures may be frequently involved.

### Corticobasal Degeneration

The syndrome of CBD can have a diverse presentation and is, therefore, difficult to diagnose. Increasingly it is thought that CBD is not a singular disease, but may stem from various etiologies and present in a variety of ways. Symptoms may include asymmetric parkinsonism, apraxia, rigidity, and the infamous “alien limb” syndrome (105). Eye movement abnormalities are present in about 33% of patients at diagnosis, and involve up to 60% of cases throughout the disease course (106). Saccadic apraxia manifests as increased latency and difficulty initiating saccades, and an increase in anti-saccade errors (107, 108). This is often asymmetric, like the other motor findings of CBD.

Unfortunately, unlike Parkinson’s disease, patients with CBD tend to have a poor response to levodopa (105, 109). Currently, no disease-modifying therapies exist. Supportive treatments that have been used to alleviate symptoms include intramuscular botulism toxin and benzodiazepines for dystonia and myoclonus (110). Given the prevalence of eye movement findings in CBD, care of these patients should include attention to visual symptoms and appropriate supportive treatment.

### Dementia With Lewy bodies

Dementia with Lewy bodies is a particularly vicious form of dementia in which affected individuals suffer from progressive memory loss, visual hallucinations, and parkinsonian motor features. Studies of eye movements in DLB have shown that, like in Parkinson’s disease, these individuals tend to have increased saccade latency, reduced saccade velocity, and an increase in variability of saccades (111, 112). In addition, there has been a case report of a patient with supranuclear gaze palsy initially misdiagnosed as PSP (113). However, there is little known about how vergence is affected in DLB. Future studies of oculomotor findings in DLB should include diplopia and vergence abnormalities to assess if these disturbances are as common in DLB as they are in Parkinson’s disease.

## Conclusion

The presence of new-onset strabismus in an adult can range in severity from mild to debilitating and merits consideration of an underlying neurodegenerative disorder. Strabismus is a common finding in Parkinson’s disease and can present as diplopia or difficulty reading. It has been found to correlate with overall disease symptomatology and presents a possible biomarker for tracking disease progression. Diplopia generally responds well to treatment in Parkinson’s, although it fluctuates with dopamine dosage, which can present a challenge in management. Strabismus is also a common finding in certain types of spinocerebellar ataxia and can aid in the clinical diagnosis of a particular SCA type. It is especially common in SCA3/MJD, where a combination of vergence insufficiency and ophthalmoplegia have been found to play a role in the pathogenesis of diplopia, offering insight into disease pathophysiology and the structures affected. However, little is known about the response of strabismus to treatment in SCA, as there is a scarcity of disease-modifying treatment. Finally, other neurodegenerative disorders, such as Huntington and atypical parkinsonian syndromes, also have well-documented eye movement effects, although there is less known about strabismus and its response to treatment in these disorders.

## Author Contributions

The authors contributed equally to this work.

## Conflict of Interest Statement

The authors declare that the research was conducted in the absence of any commercial or financial relationships that could be construed as a potential conflict of interest.
